# Untargeted metabolomic profiling of serum from client-owned cats with early and late-stage chronic kidney disease

**DOI:** 10.1038/s41598-024-55249-5

**Published:** 2024-02-27

**Authors:** Nora Jean Nealon, Stacie Summers, Jessica Quimby, Jenessa A. Winston

**Affiliations:** 1 Department of Veterinary Clinical Sciences, College of Veterinary Medicine, The Ohio State University, Columbus, OH 43210 USA; 2 Department of Clinical Sciences, Carlson College of Veterinary Medicine, Oregon State University, Corvallis, OR 97331 USA

**Keywords:** Chronic kidney disease, Biomarkers

## Abstract

Evaluation of the metabolome could discover novel biomarkers of disease. To date, characterization of the serum metabolome of client-owned cats with chronic kidney disease (CKD), which shares numerous pathophysiological similarities to human CKD, has not been reported. CKD is a leading cause of feline morbidity and mortality, which can be lessened with early detection and appropriate treatment. Consequently, there is an urgent need for early-CKD biomarkers. The goal of this cross-sectional, prospective study was to characterize the global, non-targeted serum metabolome of cats with early versus late-stage CKD compared to healthy cats. Analysis revealed distinct separation of the serum metabolome between healthy cats, early-stage and late-stage CKD. Differentially abundant lipid and amino acid metabolites were the primary contributors to these differences and included metabolites central to the metabolism of fatty acids, essential amino acids and uremic toxins. Correlation of multiple lipid and amino acid metabolites with clinical metadata important to CKD monitoring and patient treatment (e.g. creatinine, muscle condition score) further illustrates the relevance of exploring these metabolite classes further for their capacity to serve as biomarkers of early CKD detection in both feline and human populations.

## Introduction

Chronic kidney disease (CKD) is a common medical condition of cats associated with significant physiological and metabolic disturbances, including cachexia, malnutrition, deranged amino acid metabolism, and oxidative stress^1,2^. The International Renal Interest Society (IRIS) recommends staging based on disease severity (Stage 1–4) using surrogate markers of glomerular filtration rate (i.e. creatinine and symmetric dimethylarginine [SDMA])^3^. Unfortunately, caregivers of early-stage CKD cats may not detect subtle clinical signs, such as weight loss and polyuria, and cats can have only slight changes on routine laboratory tests^4,5^. Therefore, recognizing CKD in early disease stages (Stage 1 and 2), before overt clinical signs of advanced disease develop, poses a challenge for veterinarians. Timely recognition would permit more frequent monitoring and treatment earlier in the course of disease, which may slow disease progression and allow detection of disease sequelae prior to developing complications^5,6^.

Metabolites are made and used during normal cellular functions, and disease causes disruption of biochemical pathways creating specific metabolomic profiles. These profiles can be discerned by characterizing the metabolome in a biological matrix using untargeted metabolomics and comparing healthy versus diseased samples. This method has been extensively performed in people with CKD and was successfully used for biomarker discovery for early diagnosis and etiology identification^7^. Various feline untargeted metabolomics studies have been performed^8–12^. The studies used plasma^9,10^, serum^11^, feces^8,9^, and urine^12^ collected from cats with early-stage CKD. Most studies used purpose-bred research cats^8–11^ and examined dietary impacts on the metabolome^8,9,11^.

To date, no published veterinary studies compare metabolite profiles between client-owned early-stage and late-stage CKD in cats. The ability to identify metabolic drivers of early- versus late-stage CKD, despite variable environmental conditions (e.g. diet, medical treatments, household) and pathophysiological conditions (naturally-occurring CKD with and without comorbidities including chronic enteropathy, cardiomyopathy, etc.), is imperative for biomarkers to be applied successfully to diverse patient populations. The objective of this study was to identify disease- and stage-specific metabolites to improve understanding of CKD pathophysiology, particularly in early-stage disease, and thereby suggest potential therapeutic targets and biomarkers of early-stage CKD. To better define metabolic disturbances in CKD cats, we applied untargeted serum metabolomics to compare client-owned cats with early- and late-stage CKD and healthy cats.

## Results

### Clinical evaluation of healthy, early-stage and late-stage CKD cats

Of the 56 cats enrolled in this study, 25 healthy cats (median, nine years; range, 1–14 years) and 30 CKD cats (median, 14 years; range, 2.5–19 years) were included in analyses. Among CKD cats, 17 had early-stage CKD (three cats IRIS CKD Stage 1; 14 cats IRIS CKD Stage 2), and 13 cats had late-stage CKD (nine IRIS CKD Stage 3; four IRIS CKD Stage 4). One cat with Stage 4 CKD and severe azotemia (creatinine 13.1 mg/dL) was classified as an outlier upon analysis of clinical and metabolomic datasets, and it was removed from all analyses. All 55 cats included in analyses were neutered. Healthy cats (14 male, 11 female) were domestic short-, medium-, or long-haired cats (22/25), Siamese (2/25), or Himalayan (1/25). All CKD cats (17 male, 13 female) were short-, medium-, or long-haired cats. All cats except for two with CKD (one cat with early-stage and one with late-stage CKD), were fasted for at least 10 h prior to sample collection.

Physical examination and laboratory parameters for healthy, CKD Stage 1 and 2, and CKD Stage 3 and 4 cats are presented in Table 1 and demographic information for individual cats is provided in Supplementary File 1. The majority of healthy cats (68%; 17/25) had normal muscle mass; 24% (6/25 cats) had mild muscle loss and 8% (2/25 cats) had moderate muscle loss. All Stage 1 and 2 CKD cats, except one had either mild (53%; 9/17), or moderate (35%; 6/17) muscle loss. Similarly, based on recorded MCS, all cats with Stage 1 and 2 except one had either mild (33%; 4/12), moderate (42%; 5/12), or severe (16%; 2/12) muscle loss. Most cats, except two healthy and two CKD cats, had serum thyroxine concentrations measured, and all were below or within the laboratory reference interval. No cats had a history of current or past hyperthyroidism. Most cats (23/25 healthy; 29/31 CKD) had a systolic blood pressure between 120–160 mmHg with a normal direct fundic examination. One healthy cat and three CKD cats had a blood pressure between 160 and 180 mmHg with a normal fundic examination. Fecal sugar centrifugation was performed in 41/55 cats and all were negative for parasite ova. A urine protein to creatinine (UPC) ratio was performed in most healthy cats (22/25) and was normal (range, 0.06–0.29). Most CKD cats had a UPC ratio performed (25/30 cats) and only two cats had proteinuria (UPC ratio, 0.7 and 1.4). Fourteen CKD cats had renal imaging to assist with staging, which involved ultrasound for all but one cat that had abdominal radiographs. Thirteen CKD cats had findings consistent with degenerative renal disease and one Stage 2 cat (creatinine 2.4 mg/dL and USG 1.018) had normal kidneys.

**Table 1:** Patient demographics, physical examination, and laboratory variables.

Variable	Healthy (*n* = 25)	Early-stage CKD (Stages 1 and 2) (*n* = 17)	Late-stage CKD (Stages 3 and 4) (*n* = 13)
Sex	14 MC, 11 FS	9 MC; 8 FS	8 MC; 5 FS
Age (years)	9 (1–14)	14 (4–17)	13 (3–19)
Body weight (kg)	4.6 (2.8–8.1)	3.9 (3.2–6.4)	4.3 (2.4–5.8)
BCS (1–9)	5 (4–9)	5 (4–7)	5 (2–6)
MCS (0–3)*	0 (0–2)^a^	1 (0–3)^b^	1.5 (0–3)^b^
Hematocrit (%)	38 (30–47)^a^	35 (31–43)	36 (23–42)^b^
Creatinine (mg/dL)	1.4 (1.1–2.2)^a^	2 (1.3–2.7)^b^	3.7 (3.1–7.4)^c^
BUN (mg/dL)	22 (18–31)^a^	39 (22–56)^b^	55 (42–90)^c^
Total Calcium (mg/dL)	9.7 (8.8–10.4)^a^	10.1 (9.2–14.4)	10.8 (9.8–13.3)^b^
Phosphorus (mg/dL)	3.7 (2.9–4.6)^a^	3.9 (2.3–6.2)	4.1 (3.1–8.9)^b^
Potassium (mEq/L)	4.2 (3.5–5.2)	4.7 (3.7–5.3)	4.6 (2.4–5.1)
Albumin (g/dL)	3.7 (2.9–4.4)	3.5 (3.2–4.0)	3.6 (3.2–3.9)
USG	1.049 (1.038–1.073)^a^	1.018 (1.010–1.039)^b^	1.016 (1.009–1.025)^b^

Numbers outside of parentheses represent the median value for each patient group, and numbers inside of parentheses show the range. For each variable, columns within each row bearing a different superscript letter were statistically different from each other (p < 0.05). *BCS* body condition score, *BUN* blood urea nitrogen, *CKD* chronic kidney disease, *FS* female spayed, *MC* male castrated, *MCS* muscle condition score, *USG* urine specific gravity.

*MCS score: 0 = normal muscle mass; 1 = mild muscle loss; 2 = moderate muscle loss; 3 = severe muscle loss.

Most healthy cats were not receiving medications, except for topical flea and heartworm preventative (selamectin) in three cats and oral glucosamine in one cat. Eight CKD cats were on one or more medications or supplements, including aluminum hydroxide (two cats), potassium gluconate (two cats), probiotic (five cats), polyethylene glycol 3350 (two cats), topical selamectin and oral glucosamine (one cat each). All healthy cats were fed a commercial diet formulated to meet the Association of American Feed Control Officials Nutritional Profile for adult feline maintenance^13^. For CKD cats, 16 were fed one or more commercial renal therapeutic diets, ten were fed an over-the-counter diet marketed for adult or senior cats, and two were fed a combination of a renal therapeutic diet and an over-the-counter adult maintenance diet. The diet was unknown in two CKD cats.

### Healthy, early-stage CKD and late-stage CKD cats have distinct serum metabolomes

The global, non-targeted serum metabolome was evaluated in 55 cats. Table 2 shows the distribution of chemical classes, including numbers of differentially abundant metabolites when comparing the three groups using a Kruskal–Wallis test with Benjamini–Hochberg adjusted p-values. Supplementary File 2 provides fold differences and pairwise p-values for all differentially abundant metabolites between each group. Across all samples, 918 metabolites were detected and included 830 named and 88 unknown metabolites. Lipids represented ~ 40.1% of the total metabolome and accounted for ~ 43.3% of differentially abundant metabolites when comparing healthy versus early-stage CKD cats, ~ 44.6% of differences between healthy and late-stage CKD cats, and ~ 32.2% of differences between early- and late-stage CKD cats. Amino acids were the second most abundant class, comprising ~ 22.4% of the metabolome, and they accounted for ~ 25.8% of differentially abundant metabolites when comparing healthy and early-stage CKD cats, ~ 22.8% of differentially abundant metabolites when comparing healthy and late-state CKD cats, and ~ 30.5% of differentially abundant metabolites when comparing early- and late-stage CKD cats.

**Table 2:** Differentially abundant metabolites in healthy cats and cats with early- versus late-stage chronic kidney disease.

Chemical class	Healthy versus early-stage CKD (Stages 1 and 2)	Healthy versus late-stage CKD (Stages 3 and 4)	Early-stage (Stages 1 and 2) versus late-stage CKD (Stages 3 and 4)
Amino acids (206)	62 (↑ 34, ↓ 28)	100 (↑ 59, ↓ 41)	55 (↑ 35, ↓ 20)
Peptides (36)	2 (↑ 1, ↓ 1)	9 (↑ 5, ↓ 4)	4 (↑ 1, ↓ 3)
Carbohydrates (22)	5 (↑ 2, ↓ 3)	10 (↑ 6, ↓ 4)	5 (↑ 4, ↓ 1)
Vitamins and cofactors (30)	13 (↑ 4, ↓ 9)	16 (↑ 6, ↓ 10)	5 (↑ 2, ↓ 3)
Energy metabolism (9)	6 (↑ 5, ↓ 1)	8 (↑ 5, ↓ 3)	2 (↑ 0, ↓ 2)
Lipids (369)	104 (↑ 66, ↓ 38)	195 (↑ 115, ↓ 80)	58 (↑ 30, ↓ 28)
Nucleotides (51)	13 (↑ 7, ↓ 6)	26 (↑ 14, ↓ 12)	12 (↑ 8, ↓ 4)
Xenobiotics (100)	12 (↑ 8, ↓ 4)	29 (↑ 13, ↓ 16)	17 (↑ 7, ↓ 10)
Unknown and partially characterized metabolites (95)	23 (↑ 11, ↓ 12)	44 (↑ 25, ↓ 19)	22 (↑ 13, ↓ 9)
Total (918)	240 (↑ 138, ↓ 102)	437 (↑ 248, ↓ 189)	180 (↑ 100, ↓ 80)

Parentheses next to chemical class indicates total number of identified metabolites. For each comparison, numbers refer to the total number of differentially abundant metabolites (p < 0.05) when comparing each pair of treatments. Numbers in parentheses specify how many of these differentially abundant metabolites were increased (↑) in the first group relative to the second group and decreased (↓) in the first group relative to the second group. Statistical significances are based on Kruskal–Wallis testing of median-scaled log-transformed metabolite abundances, and significance was defined as p < 0.05 following Benjamini–Hochberg posthoc analysis.

*CKD* chronic kidney disease.

**Figure 1:**
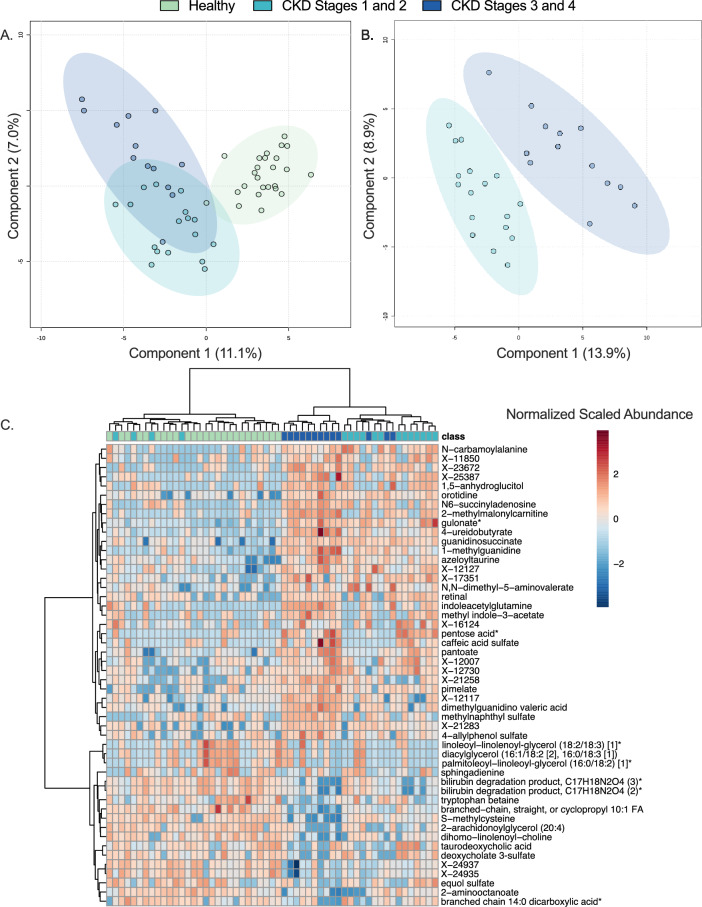
Disease stage distinctly differentiates the serum metabolome of healthy, early-stage CKD, and late-stage CKD cats. Partial least squares discriminant analysis (PLS-DA) projections of healthy cats, cats with early-stage CKD (Stages 1 and 2), and cats with late-stage CKD (Stages 3 and 4) (**a**) and cats with early-stage CKD versus late-stage CKD (**b**). Each circle represents the serum metabolome of one cat. Shaded ellipses surrounding each patient group represent 95% confidence intervals. Unsupervised hierarchical clustering analysis and heatmap of the 50 serum metabolites with the largest PLS-DA mean decrease accuracy scores (**c**). Each column represents one cat and each box represents one metabolite. Class boxes refer to the disease state of each cat, where green boxes indicate healthy cats, teal boxes represent early-stage CKD cats, and navy boxes represent late-stage CKD cats. Metabolite box colors reflect the normalized, scaled relative abundance of each metabolite when scaled across the dataset, where red boxes reflect an increased normalized abundance relative to the dataset median and blue boxes show metabolites with decreased normalized abundance relative to the dataset median. Branch points were calculated using Euclidean distances where longer branches indicate larger differences between cats. *CKD* Chronic kidney disease, *FA* Fatty acid. [1] and [2] in metabolite names are used to indicate isomers and * in names indicates metabolite identities were made using in-silico annotations.

In addition to Kruskal–Wallis testing, partial least squares discriminant analysis (PLS-DA) was used as a second multivariate metric to identify metabolites that were important contributors to explaining differences between healthy cats, those with early-stage CKD, and those with late-stage CKD. The PLS-DA model showed clear separation when comparing metabolite profiles of healthy cats to those with early-stage CKD and late-stage CKD, with 11.1% and 7.0% of metabolome differences between patient groups explained by components 1 and 2 respectively (Fig. 1A). This full PLS-DA model comparing healthy versus early-stage CKD versus late-stage CKD cats had a predictive accuracy of 0.709, a Q2 value of 0.39 and an R^2^ value of 0.39. Further separation of early-stage CKD versus late-state CKD cats was additionally observed, with 13.9% and 8.9% of metabolome differences explained by components 1 and 2 respectively (Fig. 1B). To assess major metabolite contributors to differences between healthy, early-stage CKD, and late-stage CKD cats, PLS-DA with hierarchical clustering analysis (HCA) (Fig. 1C) was used to identify metabolites that most readily discriminated between the three patient groups. Clear separation between all three groups was observed when the top 50 most discriminating PLS-DA ranked metabolites were included in the HCA projection (Fig. 1C). These metabolites included 17 unknown metabolites, 12 lipids, eight amino acids, five xenobiotics, three nucleotides, three vitamins/cofactors, one carbohydrate, and one energy metabolite.

### Lipid metabolism is a key driver of metabolome differences between healthy, early-stage CKD and late-stage CKD cats

**Figure 2:**
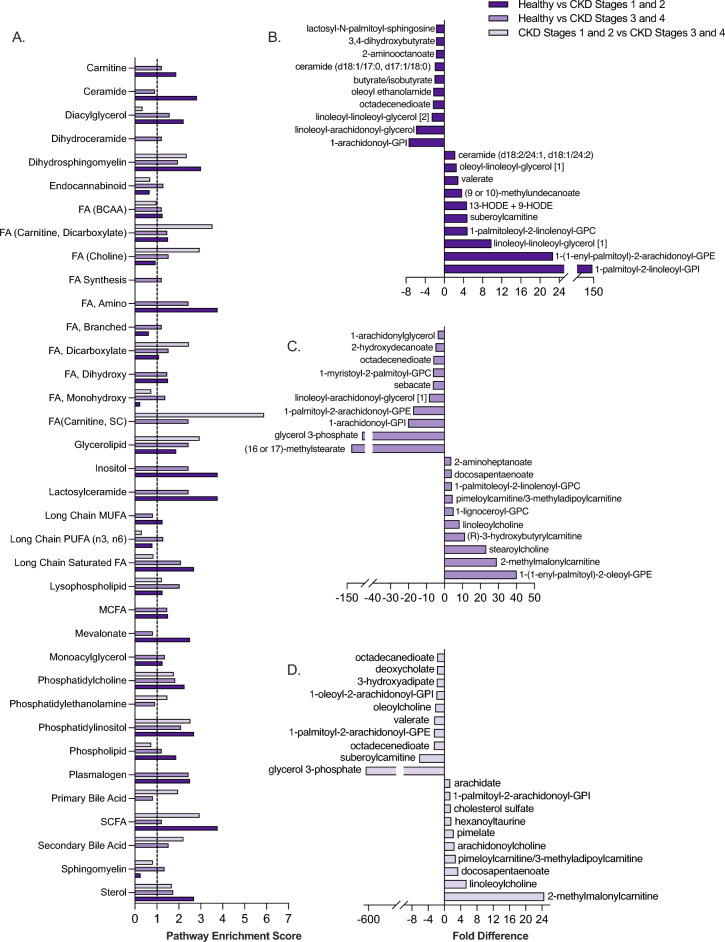
Lipid metabolism is a key driver of serum metabolome differences between healthy, early-stage CKD and late-stage CKD cats. Pathway enrichment scores of lipid metabolic pathways comparing healthy cats, early-stage (Stages 1 and 2) and late-stage (Stages 3 and 4) cats (**a**). Dotted line at 1.0 shows metabolic pathways that were defined as meaningful contributors to patient group differences (pathway enrichment score of ≥ 1.0 in at least one patient group). Differentially abundant serum lipids with the 20 largest fold differences when comparing healthy cats versus early-stage CKD cats (**b**), healthy cats versus late-stage CKD cats (**c**) and early-stage CKD versus late-stage CKD cats (**d**). Significance was defined as p ≤ 0.05 following Benjamini–Hochberg adjustments to a Kruskal–Wallis test comparing normalized, scaled abundances of each metabolite across the three patient groups. *BCAA* Branched-chain amino acid, *CKD* Chronic kidney disease, *FA* Fatty acid, *GPC* glycerophosphorylcholine, *GPE* glycerophosphorylethanolamine, *GPI* glycerophosphorylinositol, *HODE* Hydroxyoctadecadienoic acid, *MCFA* Medium-chain fatty acid, *MUFA* Mono-unsaturated fatty acid, *SC* Short-chain, *SCFA* Short-chain fatty acid. [1] and [2] in metabolite names are used to indicate isomers.

Lipids, which comprised most of the serum metabolome in healthy, early-stage, and late-stage CKD cats, were examined further to identify the metabolic pathways and metabolites contributing to the largest differences between disease states (Fig. 2). Given the diversity of lipids and metabolic pathways within this chemical class, pathway enrichment scores (PES) were used to identify key lipid pathways and metabolites contributing to differences between patient groups. When comparing healthy and early-stage CKD cats, 26 pathways were identified as significant contributors to metabolite differences (Fig. 2A). Among these metabolic pathways, fatty acid (FA) (amino and shortchain fatty acid [SCFA]) and lactosylceramide metabolism each had a PES of 3.76, which was the highest PES observed between these two patient groups. When ranking lipid metabolites by their magnitude of fold difference between healthy and early-stage CKD cats, the FA metabolites 2-aminooctanoate (1.73-fold decrease in early-stage CKD versus healthy, p = 0.0053), butyrate/isobutyrate (2.05-fold decrease in early-stage CKD versus healthy, p = 4.80 E-5) and valerate (0.34-fold decrease in early-stage CKD versus healthy, p = 7.89 E-4) were among the top 20 most differentially abundant lipid metabolites between healthy and early-stage CKD cats (Fig. 2B). Of note, the phosphatidylinositol metabolism (PES 2.68) metabolite 1-palmitoyl-2-linoleoyl-GPI was 0.0069-fold decreased in early-stage CKD versus healthy cats (p = 0.035).

In healthy versus late-stage CKD cats, 31 lipid metabolic pathways were significant contributors to metabolome differences. Similar to healthy versus early-stage CKD cats, FA metabolic pathways (acyl carnitine, amino, short-chain) were also significant contributors to differences among healthy versus late-stage CKD cats (all PES 2.44). Plasmalogen and glycerolipid metabolism (both PES 2.44) and branched chain FA metabolism (PES 1.22) further contributed several of the highest-magnitude differentially abundant metabolites for healthy versus late-stage CKD cats (Fig. 2A). These metabolites included the glycerolipid glycerol-3-phosphate (57.55 fold-decrease in late-stage CKD versus healthy cats, p = 0.033), the branched fatty acid (16 or 17)-methylstearate (123.40-fold decrease in late-stage CKD versus healthy, p = 0.015), the amino fatty acid 2-aminoheptanoate (0.26-fold decrease in late-stage CKD versus healthy, p = 8.0E−4), and the plasmalogen 1-(1-enyl-palmitoyl)-2-oleoyl-GPE (0.024-fold decrease in late-stage CKD versus healthy, p = 2.0E−4) (Fig. 2C).

When comparing early-stage CKD versus late-stage CKD cats, 14 lipid metabolic pathways were significant contributors to metabolome differences. FA metabolic pathways accounted for several of these pathways and included acyl carnitine and dicarboxylate FA (PES 3.53), acyl choline (PES 2.94), and SCFA metabolism (PES 2.94) (Fig. 2A). Among the most differentially abundant lipid metabolites within these pathways included the acyl carnitine and dicarboxylate metabolite pimeloylcarnitine/3-methyladipoylcarnitine (2.77-fold increase in early-stage CKD versus late-stage CKD, p = 0.011), the acyl choline metabolite oleoylcholine (2.27-fold decrease in late-stage CKD versus early-stage CKD, p = 0.042), and the short-chain FA metabolite valerate (2.49-fold decrease in late-stage CKD versus early-stage CKD, p = 0.011) (Fig. 2D). The glycerolipid (PES 2.94) metabolite glycerol-3-phosphate was 656.88-fold decreased in late-stage CKD cats relative to early-stage CKD cats (p = 0.012) and it was the largest lipid fold difference among all lipids and group comparisons in the dataset.

### Compared to healthy cats, derangements in serum amino acids are observed in early-stage and late-stage CKD cats

**Figure 3:**
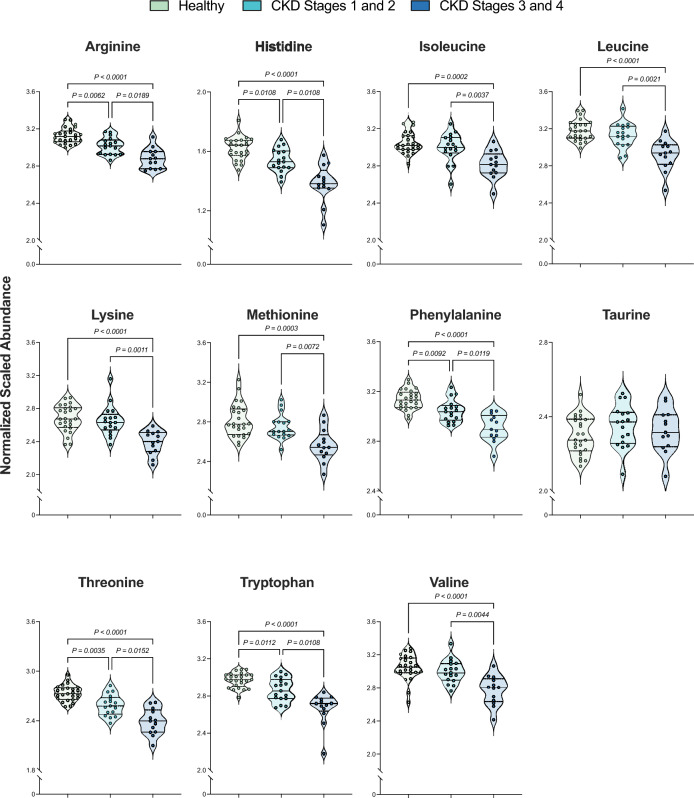
Cats with late-stage CKD exhibit decreased serum abundances of essential amino acids compared to healthy cats and those with early-stage CKD. Normalized, scaled abundances of 11 feline essential amino acids. Each circle represents one cat, where colors refer to patient groups: Green = Healthy cat; Teal = Early-stage CKD cat (Stages 1 and 2); Navy = Late-stage CKD cat (Stages 3 and 4). Dotted lines on each violin plot show the 25th, 50th (median) and 75th percentiles of normalized, scaled metabolite abundance distributions for each amino acid. Significance was defined as p ≤ 0.05 following Benjamini–Hochberg adjustments to a Kruskal–Wallis test comparing normalized, scaled abundances of each metabolite across the three patient groups. *CKD* chronic kidney disease.

Amino acids were the second largest chemical class contributing to differences between healthy and CKD cats, including between early-stage CKD versus late-stage CKD cats. Considering the prevalence of cachexia and lean muscle loss in cats with CKD^14^, differences in the 11 essential feline amino acids (EAA) were compared between groups (Fig. 3, Table 3). Five of the 11 EAAs were significantly decreased in both early-stage CKD and late-stage CKD cats versus healthy cats. This included arginine, histidine, phenylalanine, threonine, and tryptophan. Ten of the 11 EAAs were significantly decreased in late-stage CKD versus early-stage CKD. This included arginine, histidine, isoleucine, leucine, lysine, methionine, phenylalanine, threonine, tryptophan, and valine. None of the examined amino acids were significantly increased in late-stage CKD relative to early-stage CKD. One of the EAA, taurine, exhibited no differences between groups.

**Table 3:** Essential amino acid differences across healthy cats and cats with early-stage versus late-stage chronic kidney disease.

Amino acid	Early-stage CKD (Stages 1 and 2) versus healthy	Late-stage CKD (Stages 3 and 4) versus healthy	Early-stage (Stages 1 and 2) versus late-stage CKD (Stages 3 and 4)
Arginine	**↓ 0.96 *(0.0062)***	**↓ 0.92 (< *1.00E−6*)**	**↑ 1.04 (*0.018*)**
Histidine	**↓ 0.94 (*0.010*)**	**↓ 0.85** (**< *1.00E−6***)	**↑ 1.11** (***0.010***)
Isoleucine	0.98 (*0.38*)	**↓ 0.92 (*0.00015*)**	**↑ 1.06 (*0.0037*)**
Leucine	0.91 (*0.25*)	**↓ 0.91 (*0.000021*)**	**↑ 1.07 (*0.0021*)**
Lysine	0.99 (*0.47*)	**↓ 0.89 (*0.000053*)**	**↑ 1.10 (*0.011*)**
Methionine	0.91 (*0.36*)	**↓ 0.91 (*0.00033*)**	**↑ 1.07 (*0.0071*)**
Phenylalanine	**↓ 0.97 (*0.0091*)**	**↓ 0.92 (< *1.00E−6*)**	**↑ 1.04 (*0.011*)**
Taurine	1.02 (*0.36*)	0.98 (*0.50*)	1.01 (*0.50*)
Threonine	**↓ 0.94 (*0.0034*)**	**↓ 0.87 (< *1.00E−6*)**	**↑ 1.08 (*0.015*)**
Tryptophan	**↓ 0.96 (*0.011*)**	**↓ 0.89 (< *1.00E6*)**	**↑ 1.07 (*0.010*)**
Valine	0.91 (*0.24*)	**↓ 0.91 (*0.000057*)**	**↑ 1.08 (*0.0044*)**

For each comparison, numbers indicate the fold difference when comparing each group of cats, where fold differences were calculated by dividing the first group by the second group. Fold differences and statistical significances are based on Kruskal–Wallis testing of median-scaled log-transformed metabolite abundances, and significance was defined as p < 0.05 following Benjamini–Hochberg posthoc analysis. Italic numbers in parentheses represent the p-value for this test. Bold with ↑ indicates that the amino acid metabolite was significantly increased in the first group relative to the second group.

*CKD* chronic kidney disease.

### Uremic toxin metabolism differs between healthy versus CKD cats and when comparing early-stage versus late-stage CKD cats

**Figure 4:**
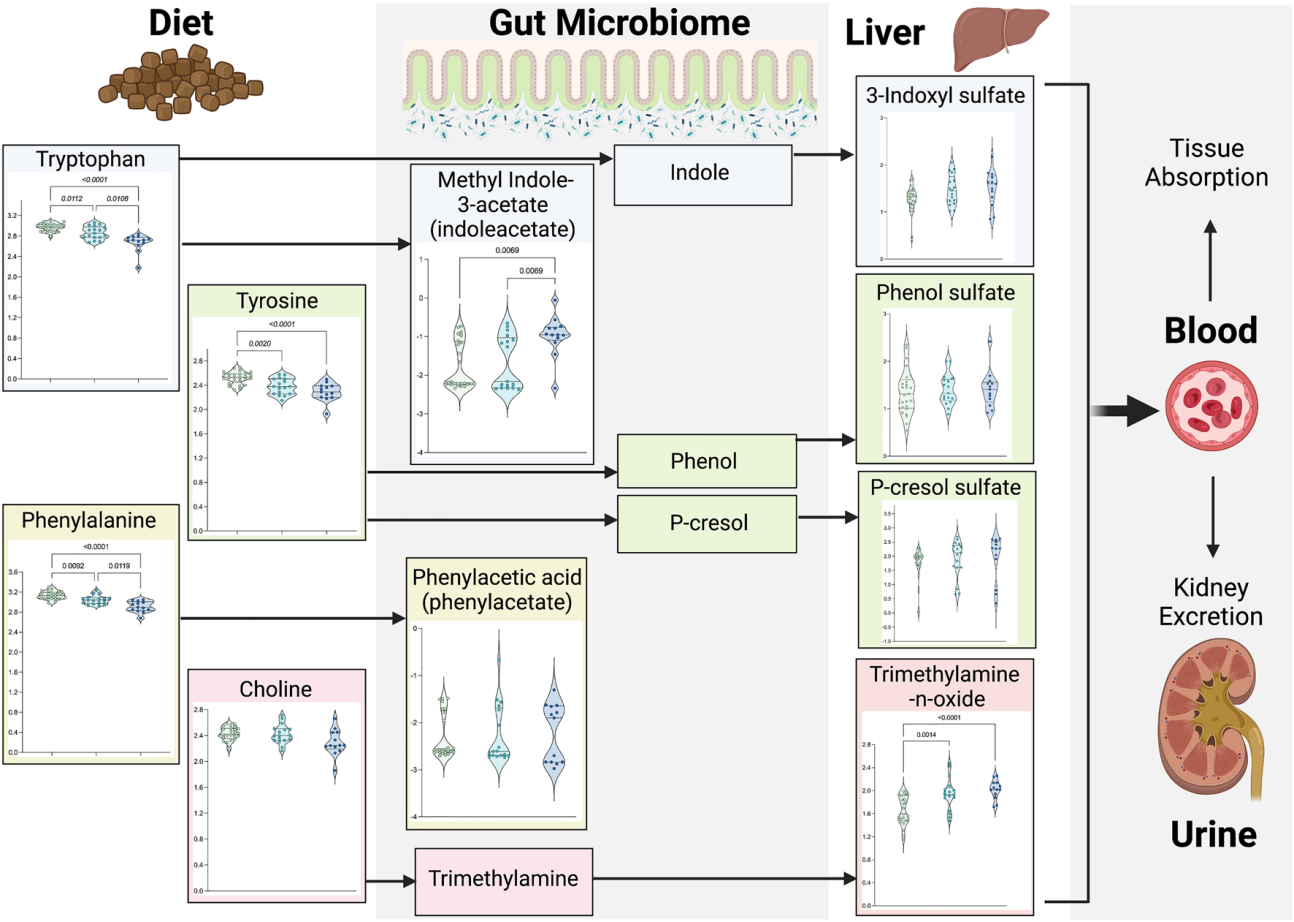
Increased abundances of uremic toxins are present in the serum of cats with late-stage versus early-stage CKD and healthy cats. Normalized, scaled abundances of ten uremic toxins. Each circle represents one cat, where colors refer to patient groups: Green = Healthy cat; Teal = Early-stage CKD cat (Stages 1 and 2); Navy = Late-stage CKD cat (Stages 3 and 4). Dotted lines on each violin plot show the 25th, 50th (median) and 75th percentiles of normalized, scaled metabolite abundance distributions for each amino acid. Arrows between metabolites indicate their relationships to each other in uremic toxin metabolic pathways, where metabolites to the left of an arrow are upstream metabolites (precursors) to the metabolites on the right side of arrows. Significance was defined as p ≤ 0.05 following Benjamini–Hochberg adjustments to a Kruskal–Wallis test comparing normalized, scaled abundances of each metabolite across the three patient groups. Figure created with BioRender.com. *CKD* chronic kidney disease.

Given the link between the gut microbiome and uremic toxins, differences in ten metabolites involved metabolism of major gut-derived uremic toxins were evaluated in Fig. 4. When comparing early-stage CKD and healthy cats, significant decreases were observed for tryptophan (0.96-fold decrease, p = 0.011), tyrosine (0.94-fold decrease, p = 0.0020), and phenylalanine (0.97-fold decrease, p = 0.0092). Tryptophan is a metabolite precursor to the uremic toxins indoxyl-3-sulfate and indoleacetate, tyrosine is the precursor for the uremic toxins phenol sulfate and p-cresol sulfate, and phenylalanine is the precursor for the uremic toxin phenylacetate. No significant differences in metabolite abundance were observed for these uremic toxins when comparing healthy versus early-stage CKD cats. When comparing late-stage CKD and healthy cats, significant decreases in tryptophan (0.89-fold decrease in late-stage CKD versus healthy cats, p < 1.00E−6), phenylalanine (0.92-fold decrease, p < 1.00E−6), and tyrosine (0.90-fold decrease, p = 0.00034) were similarly observed. Additionally, methyl indole-3-acetate was increased in late-stage CKD cats compared to healthy cats (1.81-fold increase, p = 0.0069). While there were no differences in choline abundance between groups, significant increases in the abundance of its downstream metabolite, the uremic toxin trimethylamine N-oxide (TMAO), were observed when comparing early-stage CKD to healthy cats (1.17-fold increase, p = 0.0014) and in late-stage CKD versus healthy cats (1.21-fold increase, p = 0.00010).

Disease severity further impacted uremic toxin metabolism. Compared to early-stage CKD, late-stage CKD cats exhibited significant decreases in tryptophan (0.93-fold decrease, p = 0.011) and phenylalanine (0.95-fold decrease, p = 0.012) as well as significant increases in methyl indole-3-acetate (1.72-fold increase, p = 0.0069). Interestingly, the uremic toxin 3-indoxyl sulfate (also known as indoxyl-sulfate), which when increased in serum, has previously been reported as a marker of CKD progression^15,16^, did not achieve statistical significance when comparing between early-stage versus late-stage CKD (p = 0.74).

### Correlations between selected metabolites with creatinine and muscle condition score

Associations between the 110 metabolites assessed in Figs. 1, 2, 3 and 4 and selected clinical variables were further evaluated using Spearman and Pearson correlations (Table 4, Supplementary File 3). Given their roles in GFR estimation and muscle metabolism respectively^17,18^, metabolites strongly and moderately correlated with serum creatinine and MCS were examined further (Table 4). Creatinine and MCS were not evaluated in-tandem, as independent variables of a multivariate linear regression model, because they were not normally distributed across one or more of the patient groups being examined, they were significantly-correlated with each other (r = 0.33, p = 0.014), and because MCS is known to differentially impact serum creatinine values based on the extent of a patient’s muscle wasting^4^. Seven metabolites were strongly correlated with creatinine including the vitamin C metabolite gulonate (r = 0.77, p < 1.00E−6), the nucleotides 4-ureidobutyrate (r = 0.72, p = 0.0010) and orotidine (r = 0.72, p = 0.0010), the unknown metabolite X-21283 (r = 0.71, p < 1.00E−6), the dicarboxylate FA suberoylcarnitine (r = 0.71, p < 1.00E−6), and the EAAs threonine (r = − 0.71, p < 1.00E−6) and phenylalanine (r = − 0.74, p < 1.00E−6). Eleven metabolites were significantly moderately correlated with MCS. These included the positively-correlated carbohydrate 1,5-anhydroglucitol (r = 0.63, p < 1.00E−6), the dicarboxylate FA 3,4-dihydroxybutyrate (r = 0.58, p = 0.000006), the uremic toxin trimethylamine N-oxide (r = 0.52, p = 0.000064), the unknown metabolites X-25387 (r = 0.51, p = 0.000079) and X-12730 (r = 0.51, p = 0.000087), and the vitamin C metabolite gulonate (r = 0.51, p = 0.000098). Metabolites moderately negatively correlated with MCS included the dicarboxylate FA octadecenedioate (r = − 0.53, p = 0.000036), the amino FA 2-aminooctanoate (r = − 0.53, p = 0.000044), the branched FA (9-or-10)-methylundecanoate (r = − 0.52, p = 0.000056), the EAA threonine (r = − 0.51, p = 0.000079), and the diacylglycerol linoleoyl-linolenoyl-glycerol (r = − 0.50, p = 0.00011).

**Table 4:**
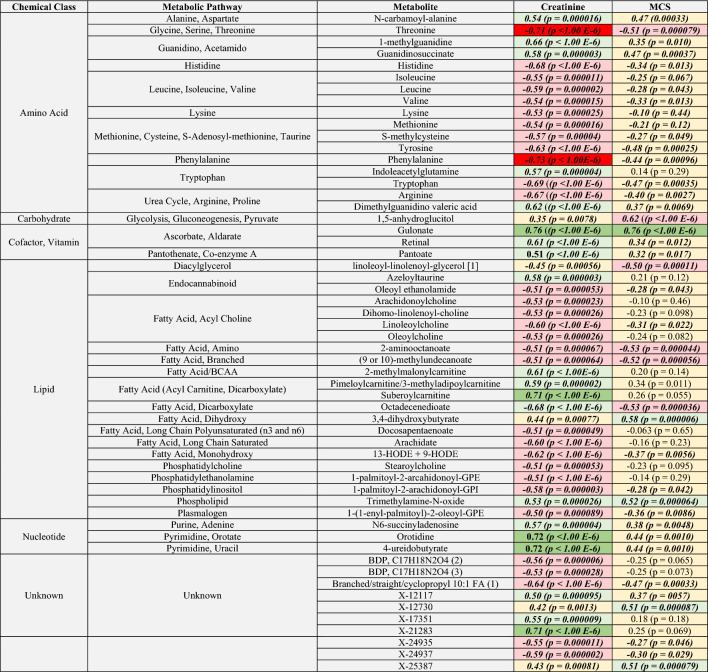
Correlations of selected metabolites with serum creatinine and muscle condition score.

Metabolites selected for correlation analysis included the 110 metabolites identified as key patient differentiators from heatmap + HCA, essential amino acid, and uremic toxin analyses. For each comparison, values show the correlation coefficient and p-value (parentheses) from Spearman’s correlations tests. Significance was defined as p < 0.05. Strongly correlated metabolites were defined as those that had a correlation coefficient between 0.70 and 0.89, and moderately-correlated metabolites included those with a correlation coefficient between 0.50 and 0.69. Box colors indicate the strength and direction of correlated metabolites where green shades indicate positive correlations (dark green = strongly correlated metabolites, light green = moderately-correlated metabolites), red shades indicate negative correlations (red = strongly correlated metabolites, pink = moderately-correlated metabolites), and beige boxes indicate metabolites that were weakly to poorly correlated with these variables. Bold and italics indicate metabolites that achieved statistical significance for the comparison of interest. *BCAA* branched-chain amino acid, *BDP* bilirubin degradation product, *FA* fatty acid, *GPC* glycerophosphorylcholine, *GPE* glycerophosphorylethanolamine, *GPI* glycerophosphorylinositol, *HODE* hydroxyoctadecadienoic acid, *MCS* muscle condition score.

## Discussion

The purpose of this study was to compare the serum metabolome of client-owned cats that were healthy to those with naturally-occurring CKD to identify metabolites that readily distinguished between health and early-stage versus late-stage disease. This is the first published study that characterizes the serum metabolome in client-owned cats. While some conventional serum biomarkers, such as creatinine and SDMA, are well correlated with GFR and routinely used for CKD diagnosis and staging, there are limitations to their clinical use. Creatinine is affected by extra-renal disorders, including lean muscle mass, endocrinopathies, diet, and it can fall within normal reference intervals during early-stage CKD^19–21^. SDMA is a more sensitive marker of GFR than creatinine and is less affected by extra-renal disorders, however mild increases can occur in the absence of kidney disease^22–24^. There is growing interest in identifying biomarkers that are highly discriminative between CKD and other diseases and that distinguish early-stage CKD from both healthy and late-stage CKD cats. Early detection could reduce disease morbidity and improve survival by improving monitoring and allowing for earlier interventions prior to the development of overt clinical signs and complications. Additionally, little is known about metabolic disturbances that occur in cats with early-stage CKD, a time when clinical signs and laboratory abnormalities can be subtle. A better understanding of these disturbances could improve understanding of disease pathophysiology and support treatments to implement early in the disease to improve outcome. Serum is a routinely obtained sample used for feline health evaluations, as well as in CKD diagnosis and staging. With the expansion of high-throughput metabolomics in veterinary medicine and the capacity of this tool to elucidate molecular mechanisms of disease, analysis of the serum metabolome represents a feasible next step to advance feline early-stage CKD biomarker discovery.

This study examined 55 client-owned cats with a confirmed diagnosis of CKD at enrollment. The significant increases observed in serum BUN and creatinine (p < 0.05 when comparing healthy versus early-stage CKD, healthy versus late-stage CKD, and early-stage versus late-stage CKD) as well as shifts in serum phosphorus, serum total calcium, hematocrit, and MCS (p < 0.05 when comparing healthy versus either early-stage or late-stage CKD) (Table 1) are similar to changes observed in feline CKD populations examined elsewhere^25^, supporting that this population is appropriately representative of client-owned cats with CKD. Within this population, distinct differences in the serum metabolome were observed when comparing healthy cats to those with early-stage and late-stage CKD (Fig. 1a,b). Similarly, Ruberti et al. showed distinct serum metabolomes between healthy and CKD Stage 1 and 2 cats, further supporting detectable metabolic disturbances in early-stage disease^11^. When comparing global metabolite differences between patient groups, lipid and amino acid metabolites contributed to the majority (> 60%) of differentially abundant metabolites between healthy and early-stage CKD, healthy and late-stage CKD, as well as between early-stage and late-stage CKD (Table 2). The importance of both lipids and amino acids in discriminating between patient groups was additionally revealed via unsupervised HCA, which showed that lipids and amino acids contributed to the majority of the metabolites that most-readily separated healthy versus CKD cats as well as between early-stage and late-stage CKD cats (Fig. 1c). Collectively, these serum metabolome changes in lipids and amino acids could be potential therapeutic targets in the management of CKD, and these chemical classes merit further investigation for biomarker discovery.

Although the roles of lipid dysmetabolism in CKD onset and progression is an active area of investigation in human medicine, little is known in dogs and cats^26^. Dogs with CKD have dyslipidemia based on lipoprotein electrophoresis^27^. Data presented herein represents the first publication to highlight systemic lipid disturbances in client-owned cats with CKD. In this study, 36 lipid metabolic pathways were found to be altered when comparing between healthy and early-stage or late-stage CKD cats and between early and late-stage CKD cats (Fig. 2a–c), where the majority of these pathways are either directly or indirectly linked to FA metabolism. Despite differences in the major etiologies of CKD between humans and cats, accumulation of lipid in the renal cortex, particularly tubular epithelium, has been documented in both people and cats with CKD^26,28^. In people, FA dysmetabolism is associated with increased FA deposition into the renal parenchyma, leading to increased inflammation and progressive tubular destruction^29^. In the current study, significant differences in the SCFAs butyrate, isobutyrate, and valerate distinguished early-stage CKD cats from healthy cats (Figs. 2a,c). In animals and people with CKD, increased butyrate, isobutyrate and valerate were associated with reduced systemic and renal pro-inflammatory cytokine production^29^, suggesting that decreased levels of these SCFAs may be early indicators of renal damage. The significant fold differences of these SCFAs when comparing healthy and early-stage CKD cats supports that these metabolites could be further examined for their capacities to identify cats with early-stage CKD and as a potential therapeutic target. Furthermore, the FA-derived metabolite glycerol-3-phosphate, which has been explored for its roles in dysregulated phosphorus metabolism and vitamin-D synthesis during renal damage^30^, was markedly decreased in late-stage CKD versus early-stage CKD, indicating that some serum FA biomarkers may decrease in abundance in the serum with advancing CKD stage, despite decreases to GFR. Although these lipid metabolites were only weakly-correlated with clinical and laboratory parameters (Supplementary File 3), the roles that lipid metabolism plays in CKD, especially in its progression from early-stage to late-stage disease, are largely unknown^26,28^, such that their levels in serum may increase or decrease via mechanisms that are not proportionate to conventional monitoring parameters. Similar to this study, robust changes to lipid metabolism were identified in the plasma metabolome of purpose-bred research cats by both Hall et al.and Jewel et al., where cats with CKD showed alterations to multiple fatty acids including phospholipids, ceramides, and dicarboxylates when compared to healthy cats^9,10^. In turn, both increases and decreases in serum lipid metabolites should be considered as biologically meaningful changes in cats with CKD.

EAAs are potential diagnostic and therapeutic biomarkers in CKD as they are easily measured and uniquely modified in CKD cats. Understanding the interrelationships between CKD and amino acid dysmetabolism is a second area of active investigation within veterinary medicine, where alterations to amino acid metabolism are linked to cachexia and uremic toxin production in feline CKD^14,31,32^. These processes are strongly tied to morbidity and mortality among CKD cats, and adjunctive therapies are routinely prescribed for these derangements during CKD management^17,33,34^, highlighting the need to better understand the molecular mechanisms driving these pathologies. CKD-induced cachexia is believed to occur due to a combination of increased nausea and appetite dysregulation, decreased total caloric and protein intake, and protein malassimilation in the gut^33^. Significant decreases in MCS were appreciated when comparing healthy cats to early-stage CKD and late-stage CKD cats (Table 1), supporting that this population of cats was ideally suited for examining serum amino acid derangements occurring secondary CKD-induced cachexia. The BCAA leucine has been widely characterized in people for its roles in protein turnover and muscle synthesis, and it decreases with cachexia and sarcopenia in elderly adults^35^, making it a promising biomarker to evaluate for early-onset cachexia that may be occurring with feline CKD. In this study, leucine was not significantly correlated with muscle condition, and thus additional exploration would be necessary to determine if it has value as a biomarker of feline cachexia. However, there were no differences in leucine abundance when comparing early-stage and late-stage CKD cats, suggesting that it also may not be suitable as an early-stage feline CKD serum biomarker.

Decreases in five amino acids were identified in the serum of cats with early-stage CKD versus healthy cats (arginine, histidine, phenylalanine, threonine, tryptophan) (Fig. 3, Table 3). These amino acids were further decreased when comparing late-stage CKD to early-stage CKD cats, suggesting that there may be progressive changes occurring with disease advancement. In a previous investigation, serum phenylalanine, tryptophan, and threonine levels decreased proportionately with CKD stage^14^. In addition to decreasing proportionately with CKD stage, threonine was moderately negatively correlated with MCS (Table 4). As an EAA, threonine in cats is an important source of cellular glucose, as an energy source for gut microbes, and as a major contributor to proteins critical for muscle development, immunity, and gastrointestinal health^36,37^. In states of decreased threonine intake, lean muscle catabolism is one method that increases threonine bioavailability to carry out these vital processes^36^, thereby explaining one mechanism for why it may decrease proportionately to MCS during feline CKD. In a study assessing dietary amino acid supplementation in cats with CKD Stage 1 and 2, threonine intake was positively-correlated with lean body mass changes^32^.

The European Uremic Toxin Work Group identified > 100 uremic toxins that are classified as either free water-soluble low molecular weight (< 500 Da), middle molecules (≥ 500 Da), or protein-bound^38^. Some major protein-bound uremic toxins originate from gut microbial metabolism of aromatic amino acids including indoxyl sulfate, p-cresol sulfate, phenol sulfate, indolacetate, and phenylacetate. Trimethylamine N-oxide is a free water-soluble low molecular weight uremic toxin that is formed in the gut via microbial fermentation of choline, phosphatidylcholine, L-carnitine, and betaine^39^. These uremic toxins are linked to systemic inflammation and oxidative stress, cardiovascular disease, immunodeficiency, cachexia, intestinal barrier damage and endotoxemia, and renal injury^40^. In this study, significantly increased abundances of indolacetate and TMAO differentiated early-stage CKD from both late-stage CKD and healthy cats (Fig. 4). TMAO was moderately positively correlated with creatinine and MCS (higher MCS indicates more muscle wasting). The relative abundance of indoxyl sulfate did not change between the three groups (Fig. 4), which differs from previous publications showing higher serum concentrations in CKD versus healthy cats^31,41^. It is important to note that these previous studies utilized targeted metabolomics to compare absolute concentrations of indoxyl sulfate to a purified standard^31^, versus the global-non-targeted approach herein using relative abundances normalized across a matrix of several hundred metabolites. However, in a non-targeted plasma metabolomics study conducted in purpose-bred research cats by Hall et al*.*, indole-3-acetate abundances did not change in cats with CKD or in healthy cats following supplementation with eight weeks of daily dietary betaine and prebiotics, meant to modulate and decrease the production of gut microbiota-derived uremic toxins^9^. Consequently, these different metabolomic detection methods should be considered when comparing shifts in uremic toxin abundances and/or concentrations across studies. Collectively, these results support that monitoring serum uremic toxin levels across disease stages can be linked to clinically-meaningful parameters that can be monitored for earlier intervention to abate disease progression.

The strengths of this study include applying a global metabolomics approach to healthy client-owned cats and those with both early-stage and late-stage CKD using serum, which is a routinely-used and non-invasive diagnostic sample. While this study was not sufficiently powered to compare cats between separate IRIS stages or sub-stages due to low enrollment of CKD Stage 1 and 4 cats, separation of cats into early-stage CKD (Stages 1 and 2) and late-stage CKD (Stages 3 and 4) groups still allows for broader comparisons; this grouping has been applied previously to compare CKD cats^14^. The diversity of diets, medications, and supplements consumed likely contributes to metabolome variation, due to differential effects on host and gut microbial metabolism^42^. The impact of these factors on metabolite abundances cannot be readily discerned in the current study, as specific formulations, frequency and duration of use are not known for all study cats. Two cats were not fasted before serum collection, which may contribute to some metabolome variation (Supplementary File 1). However, despite this, the serum metabolome robustly distinguished between healthy, early-stage CKD, and late-stage CKD cats, highlighting the clinical relevance of applying this matrix to diverse, client-owned populations of cats and/or human populations. Furthermore, the stage of disease in CKD cats was based on serum creatinine concentrations, which are negatively correlated with muscle mass^43^. This potentially impacted the disease stage cats were given.

The serum metabolome readily differentiates healthy cats from those with early-stage and late-stage CKD and supports that substantial metabolic derangements occur in early-stage CKD. Lipid and amino acid metabolites are key determinants of disease severity and are generally well-correlated with clinical variables used to guide CKD clinical decision-making CKD. Therefore, there is immense promise in investigating if these metabolites can serve as discriminative and feasibly measured biomarkers that distinguish healthy versus early-stage CKD. Given the similarities between feline and human CKD, biomarkers identified for feline early-stage CKD detection may advance human diagnostics, resulting in improved life quality for animals and people with CKD.

## Methods

### Study population

The study included serum samples from 55 cats sourced from previous studies. All studies were approved by either the Clinical Review Board (VCS 2018-168; VCS 2019-198) at Colorado State University or the Institutional Animal Care and Use Committee (IACUC) at Oregon State University (IACUC 2020-0069; IACUC 2020-0065) and were performed in accordance with relevant guidelines and regulations. Healthy cats and cats diagnosed with CKD were recruited from clients of the Colorado State University Veterinary Teaching Hospital from 2018 to 2019 and Oregon State University Veterinary Teaching Hospital from 2020 to 2021. Healthy cats were recruited specifically for these CKD clinical trials ongoing at Colorado State University or Oregon State University and performed screening at their respective hospitals. To be eligible for inclusion, cats underwent a thorough evaluation that included a client history, review of past medical record, and physical examination performed by a board-certified internal medicine specialist. Cats had a complete blood count, serum biochemistry panel, urinalysis and in most cases, urine protein-to-creatinine (UPC) ratio, fecal sugar centrifugation parasite screen, serum total thyroxine concentration, and blood pressure measurement by Doppler sphygnomanometry was performed. A nine-point body condition score (BCS; Nestle Purina, St. Louis, MO, USA) and MCS was obtained (0 = normal; 1 = mild muscle loss; 2 = moderate muscle loss; 3 = severe muscle loss)^44,45^. Cats were considered healthy based on unremarkable medical history including receiving no medications (besides flea and tick preventatives), physical examination, and normal laboratory testing, including a serum creatinine concentration within reference interval and a USG > 1.035. The reference interval for serum creatinine concentration at Colorado State University Veterinary Diagnostic Laboratory (Roche Cobras c501 Chemistry Analyzer) and the Oregon Veterinary Diagnostic Laboratory (Beckman Coulter AU480 Chemistry Analyzer) is 0.8–2.4 mg/dL and 0.6–2.0 mg/dL, respectively. The diagnosis of CKD in cats was confirmed and cats with CKD were staged by a board-certified small animal internal medicine specialist using International Renal Interest Society (IRIS) guidelines^3^. Cats with CKD were staged (CKD Stage 1–4) based on serum creatinine concentrations (Stage 1: < 1.6 mg/dL; Stage 2: 1.6–2.8 mg/dL; Stage 3: 2.9–5.0 mg/dL; Stage 4: > 5.0 mg/dL). The diagnosis of CKD in cats with Stage 1 CKD and early (non-azotemic) Stage 2 CKD was based on either ultrasound findings consistent with degenerative renal disease and/or persistent inadequate urinary concentrating ability without identifiable non-renal cause. Exclusion criteria included systemic disease including diabetes mellitus, hyperthyroidism, liver disease, known or suspect gastrointestinal disease and antibiotic therapy within the past two weeks.

### Sample collection

Sera were collected as part of unrelated studies. All participating cat owners provided written consent prior to sample collection. Serum samples were collected via jugular or medial saphenous venipuncture, frozen on site, and stored at − 80 °C within 4–6 h of collection until analysis. These samples were stored for up to three years prior to utilization in the present study.

### Metabolome sample preparation

Analysis of global serum metabolic profiles was performed by a commercial laboratory (Metabolon Inc., Morrisville, NC) as previously described. Briefly, serum was submitted for each patient on dry ice. Upon arrival, samples were stored at − 80 °C until processing. Ice cold methanol (80% methanol v/v solution maintained at − 80 °C) was added to each sample to precipitate proteins and release small molecules prior to chromatographic injection. The resultant samples were vortexed for two minutes (Glen Mills GenoGrinder 2000), centrifuged, and place on a TurboVap (Zymark) to remove any remaining ice-cold methanol solvent. Each sample was next subdivided into five separate aliquots for ultra-high performance liquid chromatography tandem mass spectrometry (UPLC-MS/MS) analysis. These aliquots included: two aliquots for reverse-phase UPLC-MS/MS with positive electrospray ionization (ESI), one aliquot for reverse-phase UPLC-MS/MS with negative ESI, a fourth aliquot for hydrophilic interaction liquid chromatography tandem mass spectrometry (HILIC/UPLC-MS/MS) with negative ESI, and a fifth aliquot saved as a spare. All aliquots were stored under nitrogen prior to downstream analysis.

### UPLC-MS/MS analysis

Each aliquot was dried and reconstituted in solvents optimized for one of four chromatographic extraction methods. This setup included one aliquot optimized for elution of hydrophilic metabolites that was gradient-eluted through a C18 column (Waters UPLC BEH C18-2.1 × 100 mm, 1.7 µm) with a mobile phase of water and methanol with 0.05% perfluoropentanoic acid and 0.1% formic acid. The second aliquot was optimized for hydrophobic metabolite extraction through the same C18 column with a gradient elution in methanol, acetonitrile, and water with 0.05% perfluoropentanoic acid and 0.01% formic acid. The third aliquot was optimized for basic metabolite extraction, through a separate C18 column, via gradient elution through a mobile phase of water and methanol titrated to a pH of 8 with 6.5 mM ammonium bicarbonate. The fourth aliquot was optimized for elution through a HILIC column (Waters UPLC BEH Amide 2.1 × 150 mm, 1.7 µm) with a mobile phase gradient of water and acetonitrile titrated to a pH of 10.8 with 10 mM of ammonium formate. Following chromatographic extraction, eluted metabolites from each sample underwent fragmentation using a heated electrospray ionization (HESI-II) source ran in either positive or negative ion mode. The first two aliquots were analyzed using positive ion mode conditions and the last two aliquots were analyzed using negative ion mode conditions. Metabolite detection was performed using a Thermo Scientific Q-Exactive high resolution/accurate mass spectrometer integrated with an Orbitrap mass analyzer set to a 35,000-mass resolution, which alternated between MS and data-dependent MS^n^ scans using dynamic exclusion. The scan range for this machine covered mass to charge ratios (m/z) of ~ 70–1000 m/z.

For all analyses, a blank was included in each sample run, where initial extraction of ultra-pure water was used to establish the baseline charge signal running through the mass spectrometer. Positive controls included external standards comprised of well-characterized human plasma samples previously run on the chromatography and mass spectrometry equipment as well as a pooled sample consisting of equal amounts of each experimental sample. A third control consisted of an internal standard cocktail that consisted of metabolites at known concentrations that would not interfere with endogenous metabolite analysis (i.e., metabolites not found in serum). Negative controls included aliquots of the solvents used in the various sample extractions. To control for chromatographic drift, sample elution through the chromatographer was randomized and control samples were proportionately spaced between experimental sample injections.

### Data extraction, compound identification and quantitation

Mass spectral data was extracted and processed using proprietary technologies owned by Metabolon Inc. Briefly, mass spectral peaks are identified as compounds through peak comparison to an internal library of over 4,500 purified standards and recurrent unknowns (i.e., metabolites identified only to the m/z level). Compound identification was made based on retention index matches, a mass match ± 10 ppm, and evaluation of forward and reverse MS/MS scores. To determine metabolite raw abundances, area under the curve was quantified for each peak after controlling for mass spectrometer current differences within runs and between runs. Pathway enrichment scores (PES) for selected metabolic pathways were calculated using the following equation:

$$Pathway \, Enrichment \, Score=\left(\frac{k}{m}\right)/ \left(\frac{n}{N}\right)$$

where “*k*” is the number of significant metabolites in the metabolic pathway, “*m*” is the number of identified metabolites in the pathway, “*n*” is the number of significant metabolites in the comparison of interest (e.g. healthy versus early-stage CKD, healthy versus late-stage CKD, or early-stage versus late-stage CKD), and “*N*” is the total number of metabolites in the dataset.

### Statistical analysis

For both clinical metadata and the serum metabolome, CKD cats were grouped as early-stage CKD (Stages 1 and 2) and late-stage CKD (Stages 3 and 4) for the purpose of statistical analysis. Clinical variables (bloodwork parameters, weight, BCS, MCS, age) were compared between healthy (n = 25), early-stage CKD (n = 17), and late-stage CKD (n = 13) cats using either a one-way ANOVA or Kruskal–Wallis test with either Tukey post-hoc correction or Dunn’s multiple comparison test, respectively. Normality was confirmed with Shapiro–Wilk test and evaluation of QQ plots was performed. Statistical analysis was performed in GraphPad Prism (Version 9.5.1, GraphPad Software LLC, Boston, MA).

Statistical analysis and visualization for metabolomics data was performed using Metaboanalyst 5.0 and GraphPad Prism^46^. All metabolites were normalized within Metaboanalyst by diving each raw abundance by the median raw abundance for that metabolite across the dataset, followed by log base 10 transformation. Differential abundance analysis comparing normalized abundances between healthy cats (n = 25), early-stage CKD cats (n = 17), and late-stage CKD cats (n = 13) was performed in GraphPad Prism using a Kruskal–Wallis test with a Benjamini–Hochberg adjustment for multiple comparisons. Unsupervised hierarchical clustering analysis (HCA) was performed in Metaboanalyst using the top 50 most discriminating metabolites (highest variable importance in projection scores) from partial least-squares discriminant analysis (PLS-DA). Heatmaps were generated in Metaboanalyst using PLS-DA VIP scores generated from normalized data with all features autoscaled (i.e., mean-centered and then divided by the standard deviation of each feature). Sample distances were calculated using Euclidean distances and clustered using Ward algorithms. To assess the PLS-DA model performance, the percent accuracy of the PLS-DA model at correctly classifying cats into the correct patient groups was calculated using the five largest components contributing to sample differences. Both Q2 (model predictive accuracy) and R^2^ (model goodness of fit) parameters were additionally calculated using Metaboanalyst default settings, which used the five largest components and fivefold cross validation approach to estimate these values. R code for Metaboanalyst analysis and visualization is provided in Supplementary File 4. Spearman’s correlations were used to evaluate the associations between clinical metadata parameters that were continuous or ranked ordinal variables, as well as between clinical variables of interest (creatinine, MCS). Pearson’s correlations were used to evaluate the associations between binary (yes/no) variables. Correlation strength was defined based on the following criteria^47^: Very weak correlation (r = 0.00–0.19), weak correlation (r = 0.20–0.49), moderate correlation (r = 0.50–0.69), strong correlation (r = 0.70–0.89), and very strong correlation (0.90–1.00). For all analyses, significance was defined as p < 0.05 following any post-hoc adjustments for multiple comparisons where indicated. For all statistical analyses, p-values smaller than 1.00E−6 were recorded as p < 1.00E−6.

### Supplementary Information

Supplementary Legends.

Supplementary Information 1.

Supplementary Information 2.

Supplementary Information 3.

Supplementary Information 4.

## Data Availability

Metabolomics and/or clinical metadata analyzed in this study is available by request and under the discretion of the corresponding author.

## Abbreviations

BDP: Bilirubin degradation product
BCS: Body condition score
BCAA: Branched chain amino acid
BUN: Blood urea nitrogen
CKD: Chronic kidney disease
FA: Fatty acid
FS: Female, spayed
GFR: Glomerular filtration rate
GPC: Glycerophosphorylcholine
GPE: Glycerophosphorylethanolamine
GPI: Glycerophosphorylinositol
HCA: Hierarchical clustering analysis
HODE: Hydroxyoctadecadienoic acid
IRIS: International Renal Interest Society
MC: Male, castrated
m/z: Mass-to-charge ratio
MCFA: Medium-chain fatty acid
MUFA: Mono-unsaturated fatty acid
MCS: Muscle condition score
PLS-DA: Partial least squares discriminant analysis
PES: Pathway enrichment score
SCFA: Short-chain fatty acid
SDMA: Symmetric dimethylarginine
TMAO: Trimethylamine N-oxide
USG: Urine specific gravity
UPLC-MS/MS: Ultra-high performance liquid chromatography tandem mass-spectrometry

## References

[1] Marino CL (2014). Prevalence and classification of chronic kidney disease in cats randomly selected from four age groups and in cats recruited for degenerative joint disease studies. J. Feline Med. Surg..

[2] O'Neill DG (2015). Longevity and mortality of cats attending primary care veterinary practices in England. J. Feline Med. Surg..

[3] *IRIS Staging of CKD*. http://www.iris-kidney.com/pdf/2_IRIS_Staging_of_CKD_2023.pdf. Accessed 24 Sep 2023.

[4] Bradley R (2019). Predicting early risk of chronic kidney disease in cats using routine clinical laboratory tests and machine learning. J. Vet. Intern. Med..

[5] Hall JA (2016). Positive impact of nutritional interventions on serum symmetric dimethylarginine and creatinine concentrations in client-owned geriatric cats. PLoS ONE.

[6] Perini-Perera S (2021). Evaluation of chronic kidney disease progression in dogs with therapeutic management of risk factors. Front. Vet. Sci..

[7] Benito S (2018). Untargeted metabolomics for plasma biomarker discovery for early chronic kidney disease diagnosis in pediatric patients using LC-QTOF-MS. Analyst.

[8] Hall JA, Jewell DE, Ephraim E (2020). Changes in the fecal metabolome are associated with feeding fiber not health status in cats with chronic kidney disease. Metabolites.

[9] Hall JA, Jewell DE, Ephraim E (2022). Feeding cats with chronic kidney disease food supplemented with betaine and prebiotics increases total body mass and reduces uremic toxins. PLoS ONE.

[10] Jewell DE (2022). Metabolomic changes in cats with renal disease and calcium oxalate uroliths. Metabolomics.

[11] Ruberti B (2022). Serum metabolites characterization produced by cats CKD affected, at the 1 and 2 stages, before and after renal diet. Metabolites.

[12] Kim Y (2022). In-depth characterisation of the urine metabolome in cats with and without urinary tract diseases. Metabolomics.

[13] *AAFCO methods for substantiating nutritional adequacy of dog and cat foods*, 13–24 (2023).

[14] Summers SC (2022). Serum and fecal amino acid profiles in cats with chronic kidney disease. Vet. Sci..

[15] Liao Y-L, Chou C-C, Lee Y-J (2019). The association of indoxyl sulfate with fibroblast growth factor-23 in cats with chronic kidney disease. J. Vet. Intern. Med..

[16] Chen CN (2018). Plasma indoxyl sulfate concentration predicts progression of chronic kidney disease in dogs and cats. Vet. J..

[17] Freeman LM (2012). Cachexia and sarcopenia: Emerging syndromes of importance in dogs and cats. J. Vet. Intern. Med..

[18] Paepe D, Daminet S (2013). Feline CKD: Diagnosis, staging and screening: What is recommended?. J. Feline Med. Surg..

[19] Kongtasai T (2022). Renal biomarkers in cats: A review of the current status in chronic kidney disease. J. Vet. Intern. Med..

[20] Peterson ME (2018). Evaluation of serum symmetric dimethylarginine concentration as a marker for masked chronic kidney disease in cats with hyperthyroidism. J. Vet. Intern. Med..

[21] Sagawa M (1995). Plasma creatinine levels and food creatinine contents in cats. J. Jpn. Vet. Med. Assoc..

[22] Mack RM (2021). Longitudinal evaluation of symmetric dimethylarginine and concordance of kidney biomarkers in cats and dogs. Vet. J..

[23] Hall JA (2014). Comparison of serum concentrations of symmetric dimethylarginine and creatinine as kidney function biomarkers in cats with chronic kidney disease. J. Vet. Intern. Med..

[24] Paltrinieri S (2017). Serum symmetric dimethylarginine and creatinine in Birman cats compared with cats of other breeds. J. Feline Med. Surg..

[25] Reynolds BS, Lefebvre HP (2013). Feline CKD: Pathophysiology and risk factors: What do we know?. J. Feline Med. Surg..

[26] Martino-Costa AL (2017). Renal interstitial lipid accumulation in cats with chronic kidney disease. J. Comp. Pathol..

[27] Behling-Kelly E (2014). Serum lipoprotein changes in dogs with renal disease. J. Vet. Intern. Med..

[28] Gai Z (2019). Lipid accumulation and chronic kidney disease. Nutrients.

[29] Magliocca G (2022). Short-chain fatty acids in chronic kidney disease: Focus on inflammation and oxidative stress regulation. Int. J. Mol. Sci..

[30] Simic P (2020). Glycerol-3-phosphate is an FGF23 regulator derived from the injured kidney. J. Clin. Invest..

[31] Summers SC (2019). The fecal microbiome and serum concentrations of indoxyl sulfate and p-cresol sulfate in cats with chronic kidney disease. J. Vet. Intern. Med..

[32] Hall JA (2019). Cats with IRIS stage 1 and 2 chronic kidney disease maintain body weight and lean muscle mass when fed food having increased caloric density, and enhanced concentrations of carnitine and essential amino acids. Vet. Rec..

[33] Freeman LM (2016). Evaluation of weight loss over time in cats with chronic kidney disease. J. Vet. Intern. Med..

[34] Brusach K (2023). Measurement of Ghrelin as a marker of appetite dysregulation in cats with and without chronic kidney disease. Vet. Sci..

[35] Casperson SL (2012). Leucine supplementation chronically improves muscle protein synthesis in older adults consuming the RDA for protein. Clin. Nutr..

[36] Hammer VA, Rogers QR, Freedland RA (1996). Threonine is catabolized by L-threonine 3-dehydrogenase and threonine dehydratase in hepatocytes from domestic cats (*Felis domestica*). J. Nutr..

[37] Tang Q (2021). Physiological functions of threonine in animals: beyond nutrition metabolism. Nutrients.

[38] Duranton F (2012). Normal and pathologic concentrations of uremic toxins. J. Am. Soc. Nephrol..

[39] Lim YJ (2021). Uremic toxins in the progression of chronic kidney disease and cardiovascular disease: Mechanisms and therapeutic targets. Toxins.

[40] Bhargava S (2022). Homeostasis in the gut microbiota in chronic kidney disease. Toxins.

[41] Cheng FP (2015). Detection of indoxyl sulfate levels in dogs and cats suffering from naturally occurring kidney diseases. Vet. J..

[42] Mertowska P (2021). A link between chronic kidney disease and gut microbiota in immunological and nutritional aspects. Nutrients.

[43] Hall JA (2015). Relationship between lean body mass and serum renal biomarkers in healthy dogs. J. Vet. Intern. Med..

[44] *Muscle Condition Score*. https://wsava.org/wp-content/uploads/2020/01/Muscle-Condition-Score-Chart-for-Dogs.pdf. Accessed 6 Aug 2023.

[45] *Body Condition Score*. https://wsava.org/wp-content/uploads/2020/08/Body-Condition-Score-cat-updated-August-2020.pdf. Accessed 6 Aug 2023.

[46] Pang Z (2022). Using MetaboAnalyst 5.0 for LC–HRMS spectra processing, multi-omics integration and covariate adjustment of global metabolomics data. Nat. Protoc..

[47] Krasztel MM (2022). Correlation between metabolomic profile constituents and feline pancreatic lipase immunoreactivity. J. Vet. Intern. Med..

