# Supplementary material for: Untargeted metabolomic profiling of serum from client-owned cats with early and late-stage chronic kidney disease
Source: Sci Rep. 2024 Feb 27;14:4755. doi: 10.1038/s41598-024-55249-5 (PMC10899575; doi:10.1038/s41598-024-55249-5)
Supplement: Supplementary file 1 — Supplementary Legends. [file 41598_2024_55249_MOESM1_ESM.docx]

**Supplementary Files
Supplementary Table 1:** Patient demographics, selected laboratory diagnostic values, owner-reported diets, medications, and supplements.

**Supplementary File 2**: Fold differences and p-values for differentially abundant metabolites when comparing between healthy, early-stage CKD, and late-stage CKD cats.

**Supplementary File 3:** Correlation coefficients (*r*), coefficients of determination (*R^2^*), and p-values for Spearman and Pearson correlation tests of selected metabolites with patient clinical metadata variables. For continuous metadata variables, Spearman correlation tests were used for analysis; for binary (yes/no) metadata variables, Pearson correlation tests were used.

**Supplementary File 4:** R code used for PLS-DA, heat map, and hierarchical clustering analysis visualization, generated by Metaboanalyst.
